# First person – Frederike Riemslagh

**DOI:** 10.1242/dmm.048920

**Published:** 2021-02-16

## Abstract

First Person is a series of interviews with the first authors of a selection of papers published in Disease Models & Mechanisms, helping early-career researchers promote themselves alongside their papers. Frederike Riemslagh is first author on ‘Inducible expression of human C9ORF72 36× G4C2 hexanucleotide repeats is sufficient to cause RAN translation and rapid muscular atrophy in mice’, published in DMM. Frederike conducted the research described in this article while a PhD Candidate in Prof. Dr Rob Willemse's lab at Erasmus Medical Center, Department of Clinical Genetics, Rotterdam, The Netherlands. She is now a Postdoctoral Fellow in the lab of Dr Christian Mosimann at the University of Colorado, USA, investigating genetic diseases that affect the heart and brain.


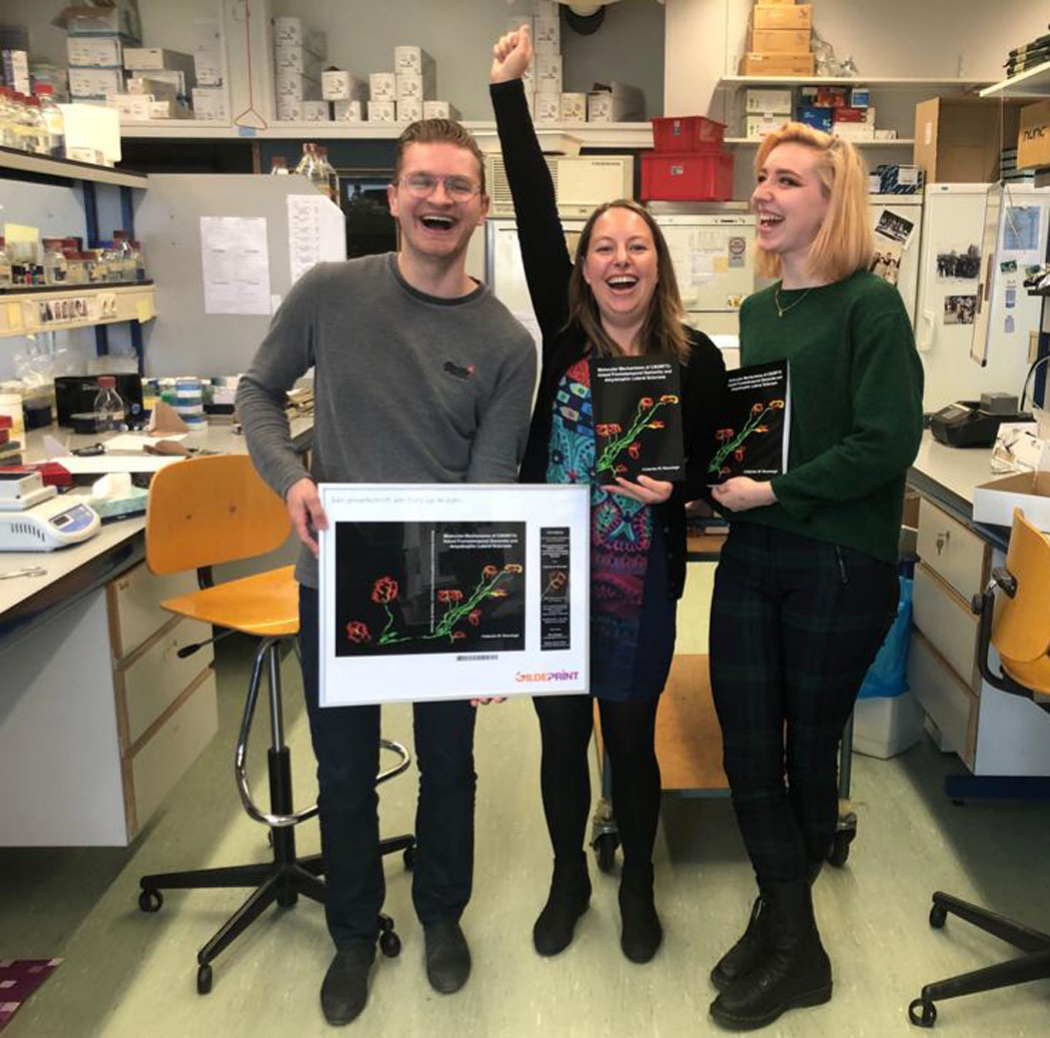


**Frederike Riemslagh**

**How would you explain the main findings of your paper to non-scientific family and friends?**

Amyotrophic lateral sclerosis (ALS) and frontotemporal dementia (FTD) are two neurological disorders that can occur together. ALS is a motor neuron disorder in which the nerve cells that innervate the muscles are affected, causing problems with walking, eating, speaking and breathing. Patients that suffer from ALS often develop problems with executive functions and memory, which are symptoms of FTD. FTD is a type of dementia in which the frontal and temporal lobe of our brain is degenerating, causing less empathy and foresight. The two diseases are genetically linked: 90% of all patients that suffer from both ALS and FTD have an alteration in their DNA; a mutation in the *C9ORF72* gene. This mutation causes the cells in our body to produce extra proteins, which are called dipeptide repeat (DPR) proteins. These proteins do not have a normal function in our body, but instead start to accumulate over time and become toxic to nerve cells. To study the toxicity of these proteins, we created a mouse model that has these extra proteins and compared them to mice without these extra DPR proteins. Mice with DPRs developed symptoms of ALS: the connections between nerves and muscles started to degenerate (see figure), they made more mistakes while walking over a horizontal ladder, and eventually their muscles were disintegrating too. To find out whether the disease could be halted, and what would be the best moment for possible treatment, we stopped DPR protein production at different timepoints during the disease. When the production of the DPR proteins was stopped before any symptoms started, the mice remained healthy. When the production of DPR proteins was stopped during the early phase of the disease, the mice still developed symptoms. Even though the cells in the body were cleared of the toxic DPR proteins, the disease still progressed. This is important information for possible new treatments; it indicates that early diagnosis and early treatment to prevent symptoms is crucial.

**What are the potential implications of these results for your field of research?**

Our study shows two things: first, the expression of only 36 C9ORF72 repeats is sufficient to produce DPRs, which upon expression evoke a clear phenotype in the mice, supporting a gain-of-function pathological mechanism of C9FTD/ALS; and second, it shows that timing of potential treatments is very important. We may have to start treatment even before the onset of any symptoms, which is only possible if we diagnose patients early on in life to prevent them from developing ALS and FTD.

**What are the main advantages and drawbacks of the model system you have used as it relates to the disease you are investigating?**

Our model allows the study of the toxicity of DPRs isolated from other disease elements, to fully understand their sole way of action, cellular targets and toxicity. Expression of DPRs alone is sufficient to drive neuromuscular junction degeneration, and many therapeutic strategies that are now developed focus on removing or blocking the production of DPRs in our body. Our results regarding treatment windows are therefore highly relevant. This specific mutation was discovered in 2011 and since then, many labs around the world have developed mouse models. Our model is certainly not the best, as it only expresses 36× repeats, whereas repeat lengths in patients are often longer. Furthermore, our mouse model does not combine the effect of C9ORF72 loss- and gain-of-function mechanisms. The best way would be to combine a C9KO mouse model with a C9BAC model to fully mimic the human situation and get the best model for studying these diseases.

**What has surprised you the most while conducting your research?**

I was not expecting that only 36 repeats would be enough for RAN translation and the production of stable DPRs. What fascinated me the most during my PhD was the discovery of RAN translation in the repeat disorder field. RAN translation stands for repeat-associated-non-AUG translation and is a newly described non-canonical way of translation. The exact conditions that trigger RAN translation, such as minimal repeat size necessary sequences and expression levels are unknown. Mechanisms like frame shifting can produce chimeric DPRs, but we have yet to discover what the contribution of all these processes is to the final pool of extra proteins in a cell. During my PhD, I made a short movie to explain RAN translation together with my colleague and fellow PhD student Saif Haify: https://www.youtube.com/watch?v=Sb9E3z0ZcIw.

**Figure:**
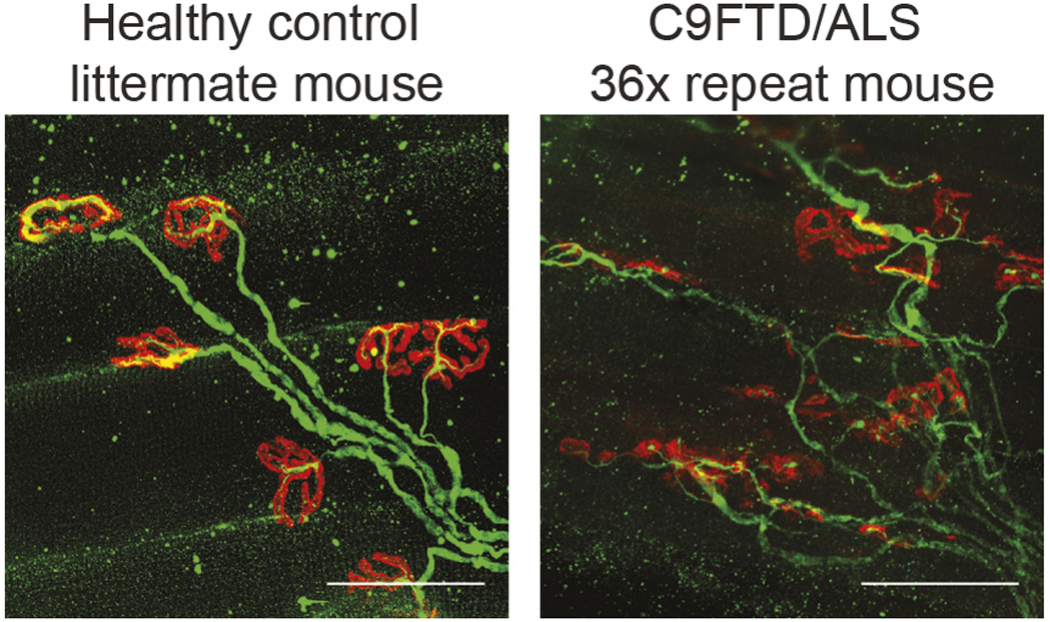
**Neuromuscular junction staining of the EDL muscle shows dissolving boutons (red, α-bungarotoxin) and disorganized axonal projections (green, neurofilament antibody) in mice with the C9ALS/FTD 36× repeat expansion compared to healthy littermate control mice.** The picture of the healthy neuromuscular junction of a control mouse was adjusted and used as cover art for my thesis! The work presented here was the main project during my PhD and could not be completed without the help of my two paranymphs, colleges and friends Rob Verhagen and Esmay van der Toorn (co-authors on the publication and also in the picture). Scale bars: 50 µm.

“What fascinated me the most during my PhD was the discovery of RAN translation in the repeat disorder field.”

**Describe what you think is the most significant challenge impacting your research at this time and how will this be addressed over the next 10 years?**

Our study indicates that the timing of treatment is important. Potential new treatments might only work or show the biggest effect when used as preventive measures. Administration of an AON drug has been proven to prevent the formation of DPRs and the development of a phenotype in a BAC mouse model of C9FTD/ALS. It is unclear whether this drug can block or reverse disease progression when it is administrated in the symptomatic phase. This brings two big challenges: first, a clinical trial on symptomatic patients might not show an effect of the drug, which might cause us to disregard its effect despite its potential in the pre-symptomatic phase; and second, pre-symptomatic carriers of the genetic mutation can be identified by genetic testing, but when and how do we decide to start preventive treatment? To identify the right moment and to test new treatments, we need clear reliable outcome measures; for example, biomarkers that measure pathological hallmarks, such as DPRs in the blood or CSF of patients. As DPRs are the targets of many new compounds, it might be possible to use them as pharmacodynamic markers of target engagement. DPR levels or other markers that remain low in pre-symptomatic carriers might prove the efficiency of a new drug. I hope we can address these points in the next ten years and enable treatment development for these two devastating diseases.

**What changes do you think could improve the professional lives of early-career scientists?**

Already early in our careers, we are often responsible for obtaining our own funding, while we do not always have a lot of first author publications. Especially during the postdoctoral phase, you are often juggling between writing grants and performing research to obtain enough results in a short timeframe, as most contracts are only for 1 to 2 years. Having more stability by providing more long-term funding and support for early researchers would allow us to focus on the research itself. Furthermore, having great mentors during these times that can help navigate the multiple phases and expectations we go through is essential. An academic career requires persistence, as your results are not always what you were hoping for, and we expect a lot of ourselves! Providing a good supporting system to build resilience is key. Find somebody who knows the research setting but is independent of your own research group to talk to and support you. Universities can help by connecting people to make young researchers less dependent on a single PI and help create a support system.

“Having more stability by providing more long-term funding and support for early researchers would allow us to focus on the research itself.”

**What's next for you?**

After receiving my PhD at the Erasmus Medical Center in The Netherlands, I moved to Denver (USA) to join the lab of Dr Christian Mosimann at the University of Colorado Anschutz Medical Campus. We use lineage tracing and genome-editing approaches in zebrafish to model human congenital diseases, and the microscopy techniques that I learn here help me to produce stunning detailed images of development! My previous training in neuroscience, along with with Christian's lab expertise on developmental and cardiovascular biology, makes the perfect combination to investigate the developmental origin and connection between the heart and the brain. My long-term goal is to establish my own independent research lab at a leading academic research institution, but right now I enjoy my time as postdoc. I am fascinated by the science and possibilities here, and love to go hiking in the Rocky Mountains at weekends!
